# The COVID-19 pandemic and its effects on dental practice at the University Clinical Dentistry Center of Kosovo

**DOI:** 10.1186/s12903-025-07292-5

**Published:** 2025-12-05

**Authors:** Agon Hoti, Ivana Sutej, Arianit Jakupi

**Affiliations:** 1 UBT- Higher Education Institution, Pristina, Republic of Kosovo; 2 University of Zagreb, Zagreb, Croatia; 3 Meditech, Pristina, Republic of Kosovo

**Keywords:** COVID-19, Pandemic, Kosovo, Dental practice, UCDCK

## Abstract

**Background:**

The COVID-19 pandemic had a profound impact on dental practices worldwide, and the University Clinical Dentistry Center of Kosovo was no exception. This study aimed to investigate the effect of the pandemic on dental practice, patient management, and antibiotic use in the absence of routine dental care. In March 2020, Kosovo’s Ministry of Health mandated the suspension of non-emergency dental services to mitigate the spread of the virus, which led to an increased reliance on antibiotics to manage dental infections remotely.

**Methods:**

This qualitative study used semi-structured interviews with 16 dentists working at UCDCK, all with more than 10 years of experience, to explore the effects of the pandemic on dental practice, patient management, and antibiotic prescribing. Interview data were thematically analyzed, and findings were supplemented with antibiotic prescription trends from 2019 to 2022.

**Results:**

During the suspension of services mandated in March 2020, the study found that 75% of dentists admitted to prescribing antibiotics as a preventive measure during the service suspension, driven by concerns about the potential for escalating infections without in-person care. Patients also exerted pressure on dentists to prescribe antibiotics during this period. The reliance on antibiotics raised concerns about antimicrobial resistance, which was exacerbated by the lack of clear prescribing guidelines. The data showed an increase in antibiotic prescriptions during the peak of the pandemic, particularly Penicillin, with a slight decline once routine dental services resumed.

**Conclusion:**

The COVID-19 pandemic disrupted dental services at UCDCK, resulting in worsened patient oral health, ethical challenges for practitioners, and increased reliance on antibiotics. These findings underscore the urgent need for clear antibiotic stewardship protocols, robust infection control measures, and the integration of tele-dentistry to improve resilience in future crises.

## Introduction

The COVID-19 pandemic has profoundly affected healthcare systems globally, with dental practices facing unique challenges due to the nature of their work. Dental procedures typically generate aerosols and involve close physical proximity, increasing the risk of viral transmission for both patients and healthcare providers. This elevated risk led to widespread changes in how dental services were delivered, and in many countries, non-emergency dental care was suspended or limited to emergency procedures to mitigate the spread of the virus [1].

During the pandemic, dental practices had to implement stringent infection control protocols, such as enhanced use of personal protective equipment (PPE), additional sanitation measures, and pre-treatment screening questionnaires to identify potential COVID-19 infections. Also, in Kosovo, there were measures taken to manage the situation. During the peak of COVID-19, the Ministry of Health of Kosovo decided to suspend all dental procedures (MoH decision 23/III/2020) due to the significantly increased risk of spreading the virus through the respiratory tract during dental procedures that generate aerosols [2]. These measures, while necessary for safety, significantly increased the cost of dental care and reduced service availability. Lewandowska [3] noted that these changes negatively affected the comfort and efficiency of dental work, contributing to higher treatment costs and limiting patient access to routine dental care. One major shift in dental practice during the pandemic was the adoption of tele-dentistry. Butt [4] highlighted that tele dentistry allowed dental professionals to offer remote consultations and triage non-emergency cases, reducing the need for in-person visits and helping maintain patient care during lockdowns. The use of telemedicine, however, was not universally accessible, with countries like China reporting significant regional disparities in the adoption of online consultations Yang [5]. The economic impact of the pandemic on dental practices was also very substantial [6] Patel [7]underscored that many dental offices experienced financial strain due to the reduction in patient visits, with some even facing the risk of closure. The suspension of non- essential services and the increased cost of infection control measures placed an immense burden on the dental sector, which, unlike other healthcare areas, saw a disproportionate decline in income. Migas [8] in Poland indicated that the increased severity of dental conditions resulted from deferred routine care. This financial hardship also affected dental education, with many institutions switching to hybrid or online learning models, which disrupted students’ practical training. Enhanced patient education regarding responsible antibiotic use and the risk of antimicrobial resistance is critical to addressing this issue Haliti [9].

The pandemic also exposed and exacerbated health inequalities, particularly in access to dental care. Vulnerable populations, such as children and the elderly, faced greater difficulties in receiving care, as public dental health programs were suspended. In England, Stennett and Tsakos [10, 11] reported that dental service use among deprived groups fell drastically during the pandemic, widening existing oral health disparities. Similarly, in Canada, Murphy [12] found that many individuals with dental needs had their appointments cancelled or delayed, leading to untreated dental issues and, in some cases, worsening health conditions.

Globally, the pandemic’s impact on dental practices paralleled the situation in Kosovo, where the suspension of routine services led to a heavier reliance on antibiotics. Similar approaches to the “3A” approach, advising, analgesics, and antibiotics, became a standard method for handling.

dental emergencies in many countries Kumar [13]. Kosovo has historically exhibited a high rate of antibiotic consumption, even before the pandemic, as documented by Jakupi [14] in a study highlighting that this elevated usage is largely influenced by prevailing practices, where antibiotics are frequently used and often serve as the first-line treatment for minor ailments such as colds, coughs, and flu-like symptoms, emphasizing that these findings underscore the critical need for effective antibiotic stewardship to mitigate the risks associated with over-reliance on pharmaceutical solutions in the absence of proper procedural interventions. Stennett and Tsakos [9, 10] noted that vulnerable populations, particularly children and the elderly, experienced reduced access to dental services during the pandemic, exacerbating oral health disparities. Similarly, Murphy [11] in Canada reported that individuals in lower-income brackets and those with pre-existing dental needs were disproportionately affected by service cancellations and delays.

The National Strategy for Antimicrobial Resistance (AMR) 2019–2021, developed by the Ministry of Health in Kosovo, outlined a framework for controlling antibiotic misuse, advocating for antibiotic stewardship in dental practices. Furthermore, the strategy’s emphasis was on cross- sectoral cooperation through the “One Health” approach. Yet, the practical implementation of these principles has been limited, as evident in the continued high rates of over-the-counter antibiotic sales (Strategic Plan for Antimicrobial Resistance, 2018) [15]. However, studies by Haliti et al. [8, 16] documented concerns about over-prescription of antibiotics in Kosovo, an issue magnified during the pandemic as patients increasingly self-medicated in the absence of regular dental services. Another study by Bajraktari and Raka [17] provides further context to the issue, highlighting that 96% of physicians in Kosovo acknowledged over-prescribing nationally, with.

60% reporting frequent patient access to antibiotics without a prescription. This accessibility contributes to the widespread misuse and development of antimicrobial resistance, a trend also noted in the dental sector. Mustafa, Islami, and Šutej [18] documented the frequent use of amoxicillin with clavulanic acid instead of amoxicillin alone, despite concerns that this combination may contribute to resistance. Recent reviews emphasize the importance of antimicrobial stewardship programs in dentistry to prevent resistance and ensure rational prescribing [19]. Bowman-Newmark et al. [20] reported that dental practices in the UK saw a surge in antibiotic prescribing due to deferred routine procedures. Similarly, Hoti [21] observed a 30% increase in antibiotic prescriptions in Kosovo from 2020 to 2021, reflecting the challenges dentists faced in managing emergencies when in-person care was limited. These findings underline the need for robust guidelines and tele-dentistry protocols to support rational antibiotic use during public health crises.

Therefore, the primary objective of this study is to gather and analyze qualitative data to gain a comprehensive understanding of the perspectives, attitudes, and experiences of key opinion leaders (KOLs) in the field of dentistry regarding the utilization of various classes of antibiotics during the COVID-19 pandemic. The research specifically focused on the Dental University Clinical Center of Kosovo as a representative case study, aiming to explore how the pandemic influenced antibiotic prescription practices, patient management strategies, and the overall adaptation of dental practices to unprecedented challenges.

The findings of this study aim to provide insights and evidence-based recommendations for managing antibiotic use in dental healthcare practices during future public health crises. These recommendations aim to support policymakers in developing frameworks for ensuring high- quality patient care while facing pandemic-related challenges.

## Methodology

A qualitative approach was employed to ensure a comprehensive analysis, integrating insights from interviews with key opinion leaders (KOLs) in the field of dentistry. Purposive sampling was used to recruit key informants with direct clinical experience at UCDCK during the pandemic. Inclusion criteria were: (1) licensed dentists employed at UCDCK during the study period; (2) at least 10 years of clinical experience; and (3) active involvement in clinical decision-making (emergency care, prescription decisions, or departmental leadership) during the COVID-19 service suspension. A total of 16 dentists participated in the study. The average age of participants was 45.6 years (SD = 6.2), with an average professional experience of 18.4 years (SD = 5.1). The sample included 10 general dentists (62.5%) and 6 oral surgeons (37.5%). All participants were full-time employees of UCDCK and actively involved in emergency patient management during the COVID-19 service suspension. Recruitment was conducted via institutional email invitations and follow-up telephone contacts until thematic saturation was achieved. Each had direct experience with emergency dental care and patient management during the COVID-19 pandemic, making them uniquely qualified to provide valuable insights into the study’s objectives.

A semi-structured interview guide was developed based on the study’s objectives and relevant literature on dental care during the COVID-19 pandemic and antibiotic stewardship. The guide covered: impacts of service suspension, triage and emergency care processes, antibiotic prescribing decisions, patient pressures, infection control practices, and preparedness recommendations. The interview guide was pilot tested with two experienced dentists (not included in the final sample) and refined for clarity. Interviews were conducted remotely (Zoom or Skype) between 2021 and 2022, lasted 45–60 min, and were audio-recorded with participants’ consent. Field notes were taken during and immediately after interviews to capture contextual details and researcher reflections. All recordings were transcribed verbatim; transcripts were anonymized and assigned unique codes (e.g., D1–D16). To triangulate qualitative findings, de-identified antibiotic prescription data for 2019–2022 were extracted from UCDCK pharmacy/clinical records (aggregate counts by month and antibiotic class). These quantitative trends were used descriptively to corroborate patterns reported in interviews (e.g., increases in specific antibiotic classes during suspension periods).

A thematic analysis approach was used following Braun and Clarke’s framework: (1) familiarization with data through repeated reading of transcripts; (2) initial coding; (3) searching for themes; (4) reviewing themes; (5) defining and naming themes; and (6) producing the report. NVivo software (QSR International) supported data organization and coding. Two researchers independently coded the first 20% of transcripts to establish a preliminary codebook and to check for consistency. Discrepancies in coding were discussed and resolved through consensus; the codebook was revised iteratively. The remaining transcripts were coded by one researcher and reviewed by a second researcher. Final themes and subthemes were agreed upon by the research team. Where relevant, qualitative findings were compared with antibiotic prescription trend data (2019–2022) to enhance interpretive validity.

To enhance credibility, member checking was performed: summary findings and selected thematic interpretations were shared with a subset of participants for confirmation and feedback. Dependability was supported by maintaining an audit trail that documented methodological decisions, codebook versions, and analytic memos. Transferability was enhanced through detailed descriptions of the study context, participant characteristics, and exemplar quotations in the results (where permitted). Reflexive notes were maintained by the lead researcher to acknowledge potential biases and to support confirmability.

Ethical approval was obtained from the Ethics Committee of the University Dental Clinical Center of Kosovo. All participants provided informed consent prior to interviews. Participants were informed of their right to withdraw at any time. Data were stored on password-protected drives, and transcripts were anonymized to maintain confidentiality. The study adhered to the principles of the Declaration of Helsinki.

Limitations include purposive sampling from a single clinical center, which may limit generalizability to other settings. Interviews relied on self-reported practices and recollection, introducing potential recall and social desirability bias. Although prescription trend data were used for triangulation, these data were analyzed descriptively and not subjected to inferential statistical testing within this qualitative study.

## Results

### Theme 1. Impact of suspension of services

The interviews with dentists highlight the negative impacts of the Ministry of Health’s COVID-19 suspension measures. Many dentists, due to their inability to provide services, reported a disruption in patient care due to postponed treatments, leading to appointment backlogs and worsening oral health for patients.

Dentists also noted the difficulty in maintaining relationships with patients, as fewer visits and uncertainty around the pandemic impacted trust and continuity of care. Further, more detailed results from the interviews will be described in the following section.

The suspension of non-emergency dental services during the COVID-19 pandemic had significant repercussions on patient care and clinic operations. All participants reported negative impacts from the suspension, with half of them describing it as a “significantly negative impact.” This indicates that the inability to provide routine dental services created substantial disruptions in maintaining patient health, as routine procedures were halted. Dentists observed an increase in untreated cases, which contributed to worsened oral health conditions and subsequently required more intensive interventions once clinics reopened. “Patients came back with more severe conditions, and what could have been treated with a filling before often required extraction after months of waiting”.

Participants were asked about the key challenges faced during suspension. The primary challenge identified by most respondents was the inability to provide services to patients. This challenge was compounded by patient reluctance to seek treatment, which some dentists attributed to fear of exposure to COVID-19 within clinical settings. Dentists also observed an increase in case complications, as untreated or delayed dental issues often escalated in severity, leading to more complex treatments upon reopening. Additionally, concerns were raised about the spread of infection due to limitations in providing comprehensive infection control measures outside routine services, which further complicated patient care.

### Theme 2. Managing emergency vs. non-emergency cases

Furthermore, the participants were asked how they managed and the frequency of the emergency and non-emergency cases. Throughout the suspension, dental practitioners continued to see emergency cases, underscoring the necessity of maintaining some level of service during public health crises. The presence of emergency cases reflects the essential nature of dental health services, even during periods of restricted operation. Interestingly, all surveyed dentists also reported instances where patients sought help for non-emergency issues, demonstrating the demand for dental care beyond the criteria of “urgent” care defined by health authorities. This highlights the challenge of managing patient expectations and needs when services are restricted, as many patients perceived their dental conditions as urgent, even if they did not strictly meet the emergency criteria.

“We received daily calls from patients with tooth pain, and many of them considered their problem an emergency, even when it wasn’t”.

### Theme 3. Patient pressure for antibiotics

Antibiotic prescription practices were also a question posed to the participants. A majority of dentists (75%) reported an increased reliance on antibiotics during the suspension period, prescribing them as a preventive measure to manage infections in the absence of in-person care. This shift in practice reflects an adaptive response to the constraints on physical interventions, as dentists aimed to mitigate infection risks remotely. However, this reliance raised concerns regarding antibiotic overuse and the potential for antimicrobial resistance. A smaller proportion (25%) opted to prescribe antibiotics only when necessary, reflecting a cautious approach influenced by clinical judgment and an awareness of the risks associated with antibiotic over- prescription.

Additionally, some dentists reported occasional pressure from patients to prescribe antibiotics, highlighting a tension between clinical judgment and patient demands.

“During the lockdown, patients insisted on antibiotics because they feared their infection would spread if they didn’t get something immediately” (D5).

### Theme 4. Antibiotic choices and prescribing practices

In terms of the question regarding the antibiotic choices and whether they felt any pressure from the patient in terms of it, the participants said that the antibiotics most frequently prescribed during this time were Penicillin’s particularly Amoxicillin. Penicillins as a broad-spectrum antibiotic, were preferred due to their effectiveness in treating common dental infections, from which Amoxiclav, a combination of amoxicillin and clavulanic acid, provided broader antibacterial coverage. Additionally, some dentists reported occasional pressure from patients to prescribe antibiotics, highlighting a tension between clinical judgment and patient demands. This underscores the need for patient education on the responsible use of antibiotics and the risks of antibiotic resistance.

Half of the respondents encountered patients diagnosed with COVID-19 who required dental services. This illustrates the continued demand for dental care among COVID-19-positive individuals, presenting unique challenges for infection control and safety within the clinic setting. The need for antibiotic treatment was also prevalent among these patients, as over half of the dentists found it necessary to prescribe antibiotics, likely to manage secondary infections or other complications associated with COVID-19.

When routine services resumed, the majority of respondents observed that patients who had postponed treatment presented with worsened conditions, necessitating more invasive interventions and antibiotic therapy. This finding highlights the risks associated with delayed dental care, as untreated conditions progress in severity. In nearly 82% of these cases, antibiotics were required to manage the infections or complications arising from deferred treatment. This shows the importance of maintaining adaptable dental services that can address critical needs even during public health emergencies to prevent deterioration of patients’ oral health.

Nearly all respondents reported documenting antibiotic prescriptions, indicating a high level of adherence to record-keeping protocols. The frequency of antibiotic prescriptions varied, with a significant proportion of patients requiring between one and five prescriptions per month. Some patients, however, required higher prescription volumes, likely due to chronic or complex conditions. This variability emphasizes the importance of monitoring antibiotic use to ensure appropriate treatment while reducing the risk of resistance.

Most dentists expressed confidence in the quality of antibiotics available in Kosovo, which they felt were effective for patient care. However, a subset expressed uncertainty or concern, underscoring the need for continued quality assurance and monitoring. The respondents also identified infection prevention and timely immunization of staff as critical strategies for future preparedness, suggesting that these measures could bolster dental institutions’ resilience during similar public health crises.

“Antibiotics became our only option when we could not intervene directly, but I was worried about resistance in the long term”.

### Theme 5. Future preparedness and strategies

The results also showed that a significant proportion of dentists demonstrated an awareness of the risks associated with unnecessary antibiotic use, with many emphasizing the need to avoid prescriptions when not clinically required. This awareness aligns with global efforts to promote antibiotic stewardship and prevent the escalation of antimicrobial resistance. The findings indicate a positive trend toward responsible antibiotic use; however, they also highlight the need for ongoing education and reinforcement of best practices to ensure adherence to antibiotic guidelines in dental settings.

Data on strategies for enhancing the preparedness of dental institutions, based on experiences during the COVID-19 pandemic, reveal key priorities among respondents. A majority (62.5%) identified robust infection prevention measures and immunization as the most effective strategies for improving readiness for similar future scenarios. These measures include stringent infection control protocols and ensuring the timely immunization of both staff and patients (Table 1).

**Table 1:**
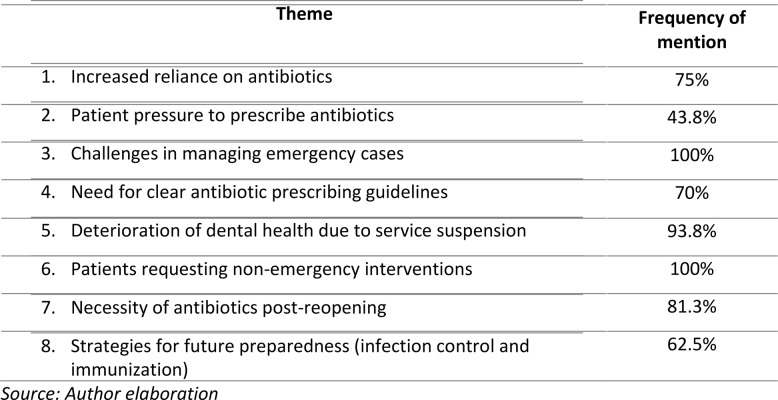
Summary of key themes from dentist interviews

The qualitative findings from the interviews revealed several key themes. The most prominent was the significant disruption caused by the suspension of dental services, which affected both dentists and patients. Dentists reported that the inability to provide routine care led to an increased reliance on antibiotic prescriptions as a preventive measure, particularly for emergency cases where infections were likely to escalate without intervention. Additionally, there was a consensus that the pandemic highlighted the need for more robust protocols to manage future public health crises, including clearer guidelines on antibiotic use.

The interview responses aligned with the data, as most participants confirmed that the increased reliance on antibiotics during the suspension of dental services was driven by the need to manage infections without the ability to provide in-person treatments. The table highlights the participants’ views on the challenges of prescribing antibiotics and the pressure they felt from patients requesting antibiotics during the pandemic.

Data on strategies for enhancing the preparedness of dental institutions, based on experiences during the COVID-19 pandemic, reveal key priorities among respondents. A majority (62.5%) identified robust infection prevention measures and immunization as the most effective strategies for improving readiness for similar future scenarios. These measures include stringent infection control protocols and ensuring the timely immunization of both staff and patients. Additionally, 25.0% of respondents emphasized the critical role of prioritizing staff immunization as a targeted approach to minimizing disease transmission in dental settings. A smaller group (12.5%) proposed focusing efforts on enhancing care exclusively during practical and support work, representing a minority perspective.

## Discussion

The main discussion for this paper will center on the consequences of the COVID-19 suspension measures on dental care, focusing on patient health, clinic operations, and healthcare practices. The suspension of routine dental services led to appointment backlogs, worsening oral health, and delayed treatments. Dentists highlighted how untreated cases escalated in severity, leading to more intensive interventions and increased antibiotic prescriptions. Suspension also led to problems in continuity of care, as dentists struggled to maintain relationships with patients. This was also described as a challenge in service delivery, or difficulty in balancing emergency cases with those that are non-emergency, which was perceived as a need for ongoing dental services at all times. The study found that 93.8% of dentists observed deteriorating dental conditions in patients due to delayed treatment, with 81.3% of these cases requiring antibiotics once services resumed. These findings align with research from other regions, such as Migas et al. (2022) in Poland, which indicated increased severity of dental conditions resulting from deferred routine care.

The findings of this study highlight that the suspension of dental services during the COVID-19 pandemic led to a marked increase in the prescription of antibiotics, particularly Penicillin, of which Amoxicillin is one of the most prescribed. Dentists in Kosovo faced multiple challenges in managing patient expectations and ensuring infection control amidst restricted physical access to dental services. This increased reliance on antibiotics underscores the need for more comprehensive preparedness plans, emphasizing clear guidelines for the use of antibiotics in dental care. During the service suspension, 75% of surveyed dentists reported prescribing antibiotics as a preventive measure, diverging significantly from their typical prescribing behavior. This adaptation was primarily motivated by the heightened risk of untreated infections. By contrast, 25% of dentists opted to prescribe antibiotics only when deemed strictly necessary, reflecting a more conservative approach. The presence of varying approaches highlights the need for clearer guidelines to help dental practitioners make consistent, evidence-based decisions. The pressure from patients also played a significant role; 43.8% of dentists reported occasional pressure from patients to prescribe antibiotics, while 12.5% felt pressured consistently. This discrepancy between patient expectations and clinical judgment further complicates antibiotic stewardship in dental practices, a consequence of low patient education, as noted by Haliti et al. (2017). While it is seen as a necessary measure to manage infections during the restriction period, concerns regarding antimicrobial resistance should be addressed.

Dentists emphasized the need for improved preparedness in future health crises, particularly in terms of infection control protocols, the adaptability of dental services, and the timely immunization of staff. This can be linked to the importance of maintaining essential services during public health emergencies to prevent further deterioration in patient health. The findings suggest a growing awareness among dentists of the risks associated with unnecessary antibiotic use, with many expressing a commitment to adhering to best practices. Ongoing education on antibiotic stewardship and its relevance to preventing antimicrobial resistance should be emphasized, alongside the need for continued monitoring of antibiotic use in dental settings.

According to the findings, the most critical strategy, identified by 62.5% of respondents, is implementing better infection prevention measures and ensuring staff and patient immunization. This highlights the importance of implementing robust infection control protocols and vaccination efforts to prevent infections. Furthermore, 25.0% of the respondents emphasized the timely immunization of staff as crucial for preventing the transmission of diseases within dental facilities. This highlights the need to ensure staff receive vaccinations promptly to minimize the risk of outbreaks. 12.5% of respondents suggested increased care, especially during practical and auxiliary work. This recommendation suggests a desire to enhance care practices during these activities, thereby better preparing dental institutions to address future challenges effectively.

## Conclusion

The COVID-19 pandemic profoundly impacted dental practice at the University Clinical Dentistry Center of Kosovo, leading to significant operational implications and changes in patient management strategies. The closure of non-emergency dental services, mandated by the Ministry of Health, resulted in an increased reliance on antibiotics as a temporary solution to manage dental infections. This reliance, while necessary during the pandemic, underscored existing challenges related to antibiotic over prescription and the lack of clear guidelines for their use in dental care. The findings of this study highlight that the suspension of routine dental services had far-reaching implications for both dentists and patients. Dentists were confronted with ethical dilemmas, balancing patient care with public health guidelines, while patients.

experienced a deterioration in their oral health due to deferred treatments. The increased demand for antibiotics during this period further emphasized the need for stronger antimicrobial stewardship and clearer prescribing protocols, as the pandemic exacerbated concerns about antimicrobial resistance in Kosovo’s dental sector. Additionally, the pandemic catalyzed the adoption of new practices, such as tele-dentistry, which helped maintain some level of patient care despite restrictions. This innovation, though not widespread in Kosovo during the pandemic, presents an opportunity for future integration into standard dental practice, especially in times of crisis. The COVID-19 pandemic not only disrupted dental services at the University Clinical Dentistry Center of Kosovo but also illuminated critical areas for improvement in dental practice management. Moving forward, a focus on improving infection control measures, enhancing antibiotic prescription with stronger guidelines, and integrating tele-dentistry could strengthen Kosovo’s dental healthcare system, ensuring better resilience in the face of future public health challenges.

## Data Availability

The datasets generated and/or analyzed during the current study are available from the corresponding author on reasonable request.

## Abbreviations

COVID-19: Coronavirus disease 2019
UCDCK: University Clinical Dentistry Center of Kosovo
MoH: Ministry of Health
PPE: Personal Protective Equipment
AMR: Antimicrobial Resistance
KOLs: Key opinion leaders
Amoxiclav: Amoxicillin with clavulanic acid
